# Fabrication of α-Fe/Fe_3_C/Woodceramic Nanocomposite with Its Improved Microwave Absorption and Mechanical Properties

**DOI:** 10.3390/ma11060878

**Published:** 2018-05-24

**Authors:** Weihong Zhou, Yunshui Yu, Xueliang Xiong, Sicong Zhou

**Affiliations:** 1 College of Material Science and Engineering, Central South University of Forestry and Technology, Changsha 410004, China; yuyunshui@sina.com (Y.Y.); wood_zhou07@sina.com (S.Z.); 2 Changsha Research Institute of Mining and Metallurgy Limited Company, Changsha 410004, China; xiongxl@minmetals.com

**Keywords:** α-Fe, Fe_3_C, woodceramic, absorption properties, mechanical properties

## Abstract

Furan resin and fir powder pretreated by FeCl_3_ and aqueous ammonia solution were used to fabricate α-Fe/Fe_3_C/woodceramic nanocomposite. The bands of the pretreated wood powder were characterized by Fourier transform infrared spectroscopy (FTIR). The structural characterization of the nanocomposites was performed by scanning electron microscopy (SEM) and X-ray diffraction (XRD). The microwave absorption of the nanocomposites was measured by a vector network analyzer in the range of 2–18 GHz. The mechanical properties of the composites were also investigated. XRD and SEM results show that the α-Fe and Fe_3_C nanoparticles are in-situ generated and disperse in the matrix of the woodceramic. The diameters of these nanoparticles increase with the increasing of concentration of FeCl_3_ solution. The experimental results show that both the complex permittivity and the complex permeability of α-Fe/Fe_3_C/woodceramic nanocomposites increase as the concentration of FeCl_3_ solution increases. The composites pretreated with 0.60 mol·L^−1^ FeCl_3_ have the best absorption properties. The maximum value of reflection loss (RL) at 3 mm thickness reaches −25.60 dB at 10.16 GHz and the bandwidth below −10 dB is about 2.5 GHz. Compared to woodceramic, the bending strength and compressive strength of α-Fe/Fe_3_C/woodceramic nanocomposites increase by 22.5% and 18.7% at most, respectively.

## 1. Introduction

Recently, microwave absorption materials have attracted much attention for their properties of eliminating or reducing the electromagnetic (EM) interference pollution and radar detection for military defense purposes. The performances of microwave absorption materials depend on their dielectric loss and magnetic loss. There have been some reports on carbon materials as dielectric loss materials including carbon black (CB), carbon nanotubes (CNTs and MWCNTs), and graphite [1,2,3].

Woodceramic can be made from various renewable materials including wood, bamboo, waste straw, bagasse, and so on. Some researches on their mechanical properties, electrical properties, friction properties, and electromagnetic shielding properties have been done [4,5,6,7]. Woodceramic is a carbon/carbon composite material that is composed of amorphous carbon from woody materials and glassy carbon from thermosetting resin. Woodceramic has low density, good conductivity and porous structure which can improve energy loss by enhancing the chance of multireflection of electromagnetic wave [8]. Consequently, woodceramic has the potential to be an absorption material. However, the mainly effect of carbonaceous material on microwave energy is dielectric loss. Ferromagnetic metal (Fe, Co, Ni and their alloys) nanoparticles have strong interaction with high frequency electromagnetic wave because of their great permeability. Accordingly, for acquiring simultaneously dielectric loss and magnetic loss, designing and fabricating a carbon/magnetic metal nanoparticles composite are interesting. Liu and co-workers [8] have reported that coconut shell charcoal/Co nanocomposite exhibited excellent wave adsorption properties. Wu and co-workers [9] have reported the evolution of the resonance absorption of bamboo charcoal/Ni_0.5_Zn_0.5_Fe_2_O_4_ composite from ferromagnetic resonance to paramagnetic resonance as temperature went through Curie point. In addition, Fe_3_C as a ferromagnetic material has excellent microwave absorption properties [10,11,12].

In this study, we propose to fabricate α-Fe/Fe_3_C/woodceramic composite to improve the microwave absorption properties of woodceramic. α-Fe nanoparticles with high chemical activities are apt to be oxidized severely in the air and they are apt to agglomerate. Therefore, α-Fe nanoparticles are obtained indirectly by using FeCl_3_ solution as precursor. The effects of concentration of FeCl_3_ on the complex permittivity and permeability of α-Fe/Fe_3_C/woodceramic composite are investigated in detail and their reflection losses (RL) are calculated. Furthermore, the effects of concentration of FeCl_3_ on the mechanical properties of the α-Fe/Fe_3_C/woodceramic nanocomposite are also discussed.

## 2. Materials and Methods

### 2.1. Material Preparation

Fir was milled into powders with a moisture content of 6% and a mean grain size of 380 μm. Furan resin was synthesized from furfural and phenol in the laboratory. The mass ratio of wood powder to furan resin is 6:4.

The wood powder was pretreated by 0.1 mol·L^−1^ NaOH solution for 12 h and then filtered and washed with deionized water repeatedly until the pH value was close to 7. The wood powder was then dipped into five FeCl_3_ solutions with the concentration of 0.1 mol·L^−1^, 0.2 mol·L^−1^, 0.3 mol·L^−1^, 0.6 mol·L^−1^ and 1.0 mol·L^−1^, respectively. After ultrasonic impregnation for 15 min, the aqueous ammonia solution was added slowly to the five FeCl_3_ solutions separately with stirring until the required pH value was obtained. After sufficient reaction, the wood powder was filtered and washed by deionized water repeatedly to remove any impurities. Lastly, the modified wood powder was obtained after drying at 105 °C for 12 h.

The modified wood powder and furan resin were thoroughly mixed and then dried at 60 °C. Subsequently, they were pressed under the pressure of 50 MPa for 25 min at 170 °C to obtain boards. The dimensions of the boards were about 100 mm × 50 mm × 25 mm. These boards were sintered in a vacuum furnace. The temperature of the furnace was raised at a heating rate of 3 °C·min^−1^ to 800 °C, keeping for 1 h at 400 °C, and keeping for 3 h at 800 °C in the heating process. The samples were coded as WCM-0.1, WCM-0.2, WCM-0.3, WCM-0.6, and WCM-1.0, respectively, where the numeral suffixes refer to the concentrations of FeCl_3_ solution impregnating wood powder.

### 2.2. Characterization

Patterns of Fourier transform infrared spectroscopy (FTIR) (8400S, Shimadzu, Kyoto, Japan) in the range of 400–4000 cm^−1^ were obtained by the KBr pellet method.

The surface morphologies and local elemental composition of the samples were observed by scanning electron microscope (SEM) (Helios Nanolab 600i, FEI, Hillsboro, OR, USA) operated at 10 kV.

XRD patterns of the samples were obtained by X-ray diffractometer (XRD) (XD-2, Beijing Purkinje General Instrument Co., Ltd., Beijing, China) with Cu-K_α_ radiation at a scanning rate of 8°·min^−1^ over an angle range of 10–90° (2θ).

The microwave absorption performances depend on electromagnetic parameters, including complex permittivity (*ε_r_*= *ε′* − *jε″*) and complex permeability (*μ_r_* = *μ′ *− *jμ″*). The electromagnetic parameters were measured by a vector network analyzer (8720ET, Agilent, Santa Clara, CA, USA) in the frequency range of 2–18 GHz. The test samples were prepared as follows: first, the samples were crushed and ground to powder with a grain size of about 74 μm, then these powders were mixed with melted wax with the weight ratio of 1:2, and finally, the mixture was placed in the steel moulds and pressed to concentric ring samples. The outer diameter was 7 mm, the inner diameter was 3.04 mm and the thickness was about 3 mm.

Using three-point bending method, the bending strength of the samples were measured by universal mechanical testing machine (MWD-50, Jinan Testing Machine Factory, Jinan, China). The crosshead speed was 2 mm·min^−1^. The dimensions of the samples were about 80 mm × 12 mm × 4 mm. Five samples were tested to obtain an average value.

The compressive strength of the samples was tested by universal mechanical testing machine (WDW-10, Jinan Xinshijin Testing Machine Co., Ltd., Jinan, China). The crosshead speed was 5 mm·min^−1^. The dimensions of the samples were about 20 mm × 10 mm × 4 mm. Five samples were tested to obtain an average value.

## 3. Results and Discussion

### 3.1. FTIR Analysis of Wood Powder

**Figure 1:**
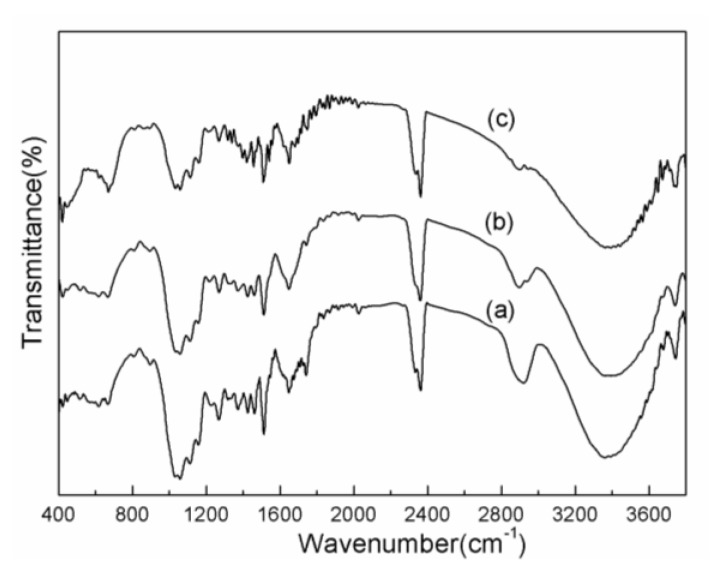
Fourier transform infrared spectroscopy (FTIR) spectra of the pretreated wood powder (**a**) wood powder without treatment; (**b**) wood powder pretreated by NaOH; (**c**) wood powder pretreated by 1.00 mol·L^−1^ FeCl_3_ and aqueous ammonia.

Figure 1 shows FTIR spectra of the pretreated wood powder. There are some bands existed in the three spectra, as shown in Figure 1a–c. For instance, the band at 3400 cm^−1^ is ascribed to stretching modes of –OH, and the band at 2920 cm^−1^ is ascribed to stretching modes of C–H. The bands at 1612 cm^−1^ and 1742 cm^−1^ are ascribe to C=O stretching modes. The band at 1512 cm^−1^ is ascribed to vibration of aromatic ring. The band at 1264 cm^−1^ is ascribed to stretching modes of C–O of esters. The band at 1059 cm^−1^ is ascribe to deformation vibration of C–O–C, and the band at 620 cm^−1^ is ascribed to external deformation vibration of O–H of alcoholic hydroxyl. By comparing Figure 1a with Figure 1b, it can be seen that the absorption peaks at 1742 cm^−1^ and 2920 cm^−1^ are weaker in the spectrum of wood powder pretreated by NaOH than in the spectrum of wood powder without treatment. The band at 1742 cm^−1^ is ascribed to C=O stretching modes, a part of which is from carboxyls of the fatty acids extracts and carbonyls of the side chain of lignin. The band at 2920 cm^−1^ is mainly ascribed to alkyl from alkane, lignin and fatty acids. It can be concluded that the fatty acids extracts in the wood have reacted with NaOH and a part of low-molecular weight lignin has been dissolved by NaOH. Consequently, wood powder has obtained more abundant pores and larger specific areas after NaOH pretreatment which is beneficial to the following penetration of FeCl_3_.

It can be observed that spectrum of Figure 1c have two absorption peaks at 420 cm^−1^ and 670 cm^−1^, corresponding to the band of Fe-OH bending vibration [13,14]. However, the two characteristic bands haven’t existed in the spectrum of Figure 1a and b. It indicates that Fe(OH)_3_ exists in the wood powder after pretreated by FeCl_3_ and aqueous ammonia solution.

### 3.2. XRD Analysis of α-Fe/Fe_3_C/Woodceramic Nanocomposites

**Figure 2:**
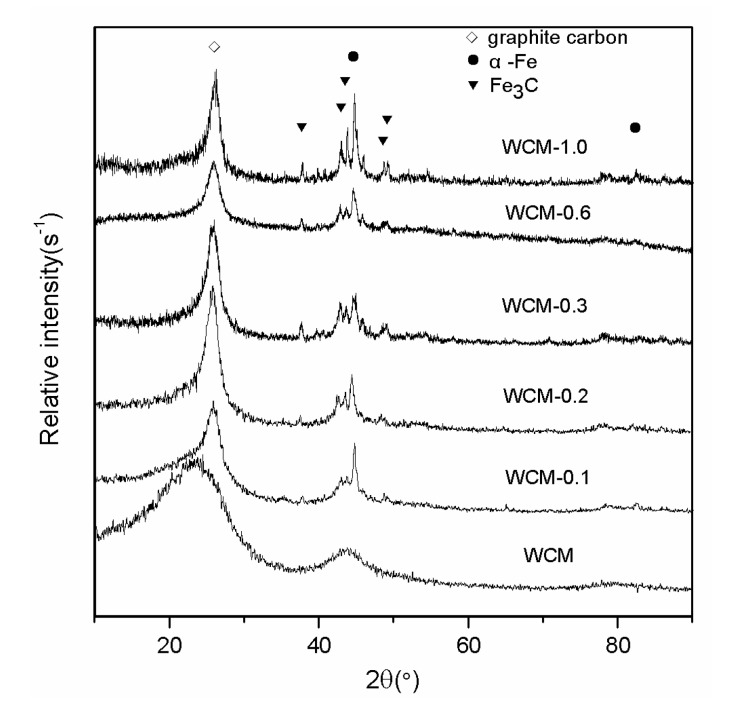
X-ray diffraction (XRD) patterns of α-Fe/Fe_3_C/woodceramic nanocomposites.

Figure 2 shows the XRD patterns of the α-Fe/Fe_3_C/woodceramic nanocomposites. The pattern of WCM represents the woodceramic without treatment by FeCl_3_ solution. The diffraction peaks appeared at 2θ = 44.6732°, 65.0211° and 82.3326° are corresponding to (110), (200) and (211) diffraction peaks of α-Fe crystal. The other diffraction peaks at around 2θ = 37.8°, 42.9°, 46.0°, 48.7° and 49.1° are attributed to the (210), (211), (112), (131), and (221) planes of Fe_3_C. The characteristic peaks of α-Fe and Fe_3_C exist in all of the patterns except for WCM. In all of the patterns it can be found the diffraction peaks of graphite carbon corresponding to (002) and (101) crystal planes. It can be observed that (002) and (101) peaks are broad and dissymmetry in woodceramic. As the concentration of FeCl_3_ increases, the peaks get more sharp and symmetric. Based on reference [15,16,17], Fe has a catalytic graphitization effect on carbon materials. Therefore α-Fe improves the extent of graphitization of woodceramic.

In the sintering process, Fe(OH)_3_ dehydrated and generated Fe_3_O_4_, and then, through a carbothermal reduction process, the Fe_3_O_4_ was reduced to Fe. The stepwise carbothermal reductions of Fe_3_O_4_ → FeO → Fe were thermodynamically favorable, respectively [18]. Furthermore, a part of Fe reacted with C to generate Fe_3_C.

### 3.3. SEM Analysis

**Figure 3:**
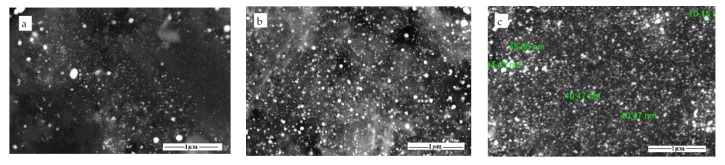

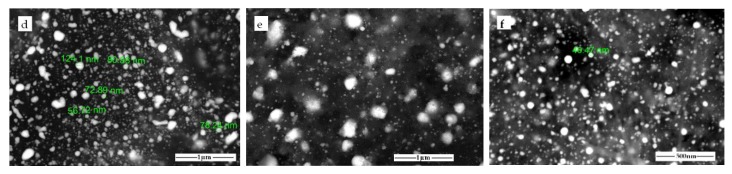
Scanning electron microscopy (SEM) photographs of α-Fe/Fe_3_C/woodceramic nanocomposites (**a**) WCM-0.1; (**b**) WCM-0.2; (**c**) WCM-0.3; (**d**) WCM-0.6; (**e**) WCM-1.0; and (**f**) WCM-0.2 with higher magnification.

Backscattered electron images of α-Fe/Fe_3_C/woodceramic nanocomposites are shown in Figure 3. It is observed that α-Fe and Fe_3_C nanoparticles are of spherical shape and well dispersed in the matrix of woodceramic. Most of these particles are lower than 100 nm when the concentration of FeCl_3_ is less than 0.60 mol·L^−1^, as shown in Figure 3a–d. Figure 3c and f show the average diameter of these particles is no more than 50 nm. As the concentration of FeCl_3_ reaches to 1.00 mol·L^−1^, it can be found some aggregates more than 100 nm as shown in Figure 3e.

### 3.4. Microwave Absorption Properties

**Figure 4:**
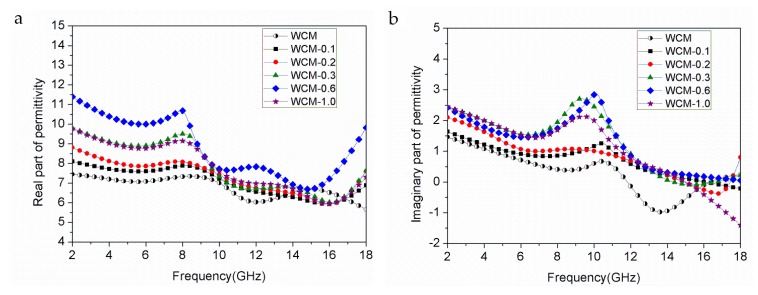
Complex permittivity of α-Fe/Fe_3_C/woodceramic nanocomposites as a function of frequency (**a**) real part of permittivity; (**b**) imaginary part of permittivity.

Figure 4 shows complex permittivity of α-Fe/Fe_3_C/woodceramic nanocomposites as a function of frequency. It can be observed that the real part of permittivity (*ε′*) and imaginary part of permittivity (*ε″*) fluctuate in the whole range of frequency. *ε′* of WCM-0.6 has the maximum value, while *ε′* of WCM has the minimum value. *ε′* represents measurement of the extent of polarization in the dynamic electromagnetic field. In the range of microwave, interfacial polarization plays a dominant role [19]. In the composite, there mainly exist four different phases including α-Fe, Fe_3_C, glassy carbon originated from furan resin and amorphous carbon originated from wood powder. The interface area exist every two of them and the sum of the interface area is very large, so interfacial polarization effects are remarkable. In addition, some defects such as dangling bands, vacancies and cavities existed in the surface of α-Fe and Fe_3_C nanoparticles lead to the change of distribution characteristic of charge. In the electric field positive and negative charges shift to the opposite direction and form electric dipole moment. As a consequent, dipole polarization also contributes to *ε′*. As the concentration of FeCl_3_ increases, interfacial polarization and dipole polarization improve. However, when the concentration of FeCl_3_ reaches to 1.0 mol·L^−1^, *ε′* begins to decrease because of a decline of the interfacial polarization caused by the aggregation of α-Fe and Fe_3_C nanoparticles.

As shown in Figure 4a, there exist two broad resonance peaks in *ε′* at about the frequency of 8 GHz and 13 GHz. This phenomenon is due to the fact that interfacial polarization is in-phase with the oscillation of the electric field vector of the electromagnetic wave. As the concentration of FeCl_3_ increases, these resonance peaks shift to the lower frequency. *ε″* represents measurement of the extent of dielectric loss in the dynamic electromagnetic field. It can be found from Figure 4b that *ε″* improves with the increasing of concentration of FeCl_3_ until the concentration of FeCl_3_ reaches to 0.6 mol·L^−1^, which has the same variation trend with *ε′*. The broad resonance peak of *ε″* is at about the frequency of 10 GHz.

**Figure 5:**
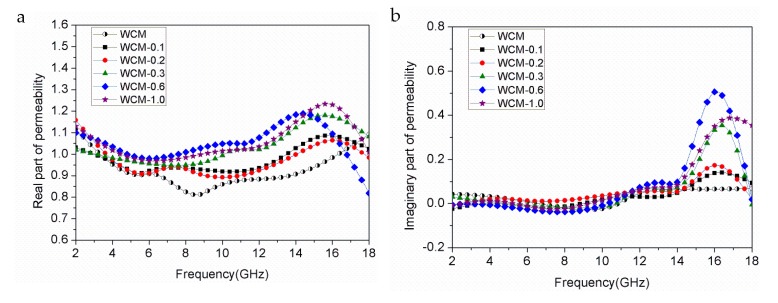
Complex permeability of α-Fe/Fe_3_C/woodceramic nanocomposites as a function of frequency (**a**) real part of permeability; (**b**) imaginary part of permeability.

Figure 5 shows complex permeability of α-Fe/Fe_3_C/woodceramic nanocomposites as a function of frequency. The real part of permeability (*μ′*) represents magnetization degree and the imaginary part of permeability (*μ″*) represents magnetic loss. It can be found that *μ′* and *μ″* fluctuate with the frequency, which indicates a phenomenon of magnetic dispersion arising from magnetization relaxation. As shown in Figure 5a, the value of *μ′* firstly decreases in the range of 2–5 GHz, then increases until getting a maximum value at about 16 GHz and lastly decreases. As shown in Figure 5b, *μ″* almost keeps constant below 0.05 with slight fluctuate in the range of 2–10 GHz. In the range of 10–18 GHz, there exist two peaks: one is at about 12 GHz and another is at about 16 GHz. These peaks at about 12 GHz are broad and peak values are low. The peak value at about 16 GHz increases with the concentration of FeCl_3_ increases. The peak values of WCM-0.1 and WCM-0.2 are relatively low and have little difference between them.

Magnetic loss may be contributed by magnetic hysteresis, eddy current loss, natural resonance, domain-wall resonance and ferromagnetic resonance. Magnetic hysteresis loss is negligible in a weak applied magnetic field. Furthermore, the value of *μ″* should be a constant when the frequency is varied if the magnetic loss only results from eddy current loss [20]. Accordingly the magnetic loss is mainly caused by ferromagnetic resonance loss. There exist effectively anisotropy fields in the α-Fe and Fe_3_C particles. When magnetic moment procession frequency is equal to magnetic field frequency of the electromagnetic wave, natural resonance occurs. The resonance peaks are ascribed to the α-Fe and Fe_3_C particles. The broad peak at about 12 GHz may be ascribed to the natural resonance of Fe_3_C particles [21]. Based on reference [22], the peak at 16 GHz is aroused from the ferromagnetic resonance of the α-Fe nanoparticles.

**Figure 6:**
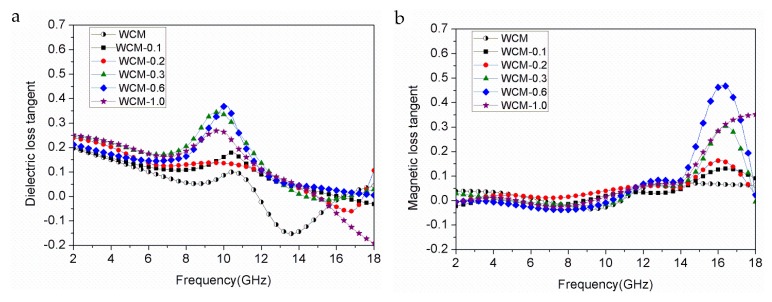
Dielectric loss tangent and magnetic loss tangent of α-Fe/Fe_3_C/woodceramic nanocomposites as a function of frequency (**a**) dielectric loss tangent; (**b**) magnetic loss tangent.

The calculated dielectric loss tangent (tg*δ_e_* = *ε″*/*ε′*) and the magnetic loss tangent (tg*δ_m_*= *μ″*/*μ′*) of α-Fe/Fe_3_C/woodceramic nanocomposites as a function of frequency are shown in Figure 6a and b, respectively. Comparing Figure 6a with Figure 6b, it can be seen both the dielectric loss tangent and the magnetic loss tangent fluctuate with the variation of frequency in the range of 2–18 GHz. The dielectric loss and magnetic loss are both lower than 1.0. The dielectric loss occupies the main position as the frequency is in the range of 2–12 GHz. The magnetic loss plays a dominant role as the frequency is in the range of 14–18 GHz. It can be observed magnetic loss tangent of WCM is close to 0, which indicates woodceramic has no magnetic loss. As the increasing of concentration of FeCl_3_ from 0.1 mol·L^−1^ to 1.0 mol·L^−1^, both the dielectric loss and magnetic loss improve.

Based on the electromagnetic parameters, the reflect loss (RL) can be calculated according to the following equation [23]:

(1) $$RL=20\log\left|\frac{Z_{in}-Z_{0}}{Z_{in}+Z_{0}}\right|,$$

where *Z*_0_ is the intrinsic impedance of free space, *Z_in_* is the normalized input impedance of the absorber and can be calculated by the following equation:

(2) $$Z_{in}=z_{0}\sqrt{\frac{\mu_{r}}{\varepsilon_{r}}}\tanh\left[j\left(\frac{2\pi fd}{c}\right)\sqrt{\mu_{r}\varepsilon_{r}}\right],$$

where *μ_r_* is the relative complex permeability, *ε_r_* is the relative complex permittivity, *f* is the frequency, *c* is the velocity of light, and *d* is the thickness of the absorber.

**Figure 7:**
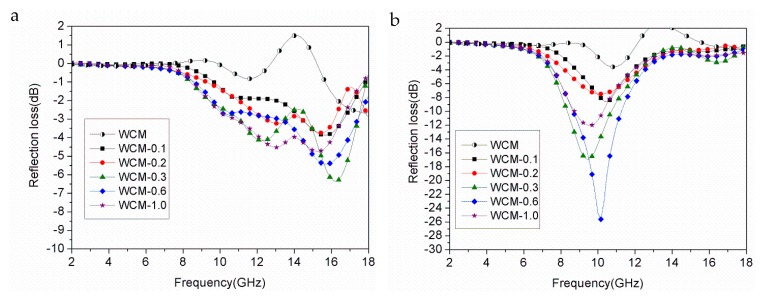
Reflection loss of α-Fe/Fe_3_C/woodceramic nanocomposites as a function of frequency (**a**) 2 mm; (**b**) 3 mm.

Figure 7 shows reflection loss (RL) at 2 mm thickness and 3 mm thickness of α-Fe/Fe_3_C/woodceramic nanocomposites as a function of frequency, respectively. The RL value of −5 dB corresponds to 70% of electromagnetic wave attenuation and the RL value of −10 dB corresponds to 90% of electromagnetic wave attenuation. The bandwidth below −5 dB of WCM-0.6 is 1.28 GHz and the bandwidth of WCM-0.3 below −5 dB is 1.44 GHz as shown in Figure 7a. It can be found the absorption properties of the composites improve as the increasing of concentration of FeCl_3_. WCM-0.6 shows the best absorption property as shown in Figure 7b. The maximum value of RL at 3 mm reaches −25.60 dB at 10.16 GHz and the bandwidth below −10 dB is about 2.5 GHz. The composite of WCM-0.3 has the second-best absorption property. Its maximum value of RL is −16.93 dB and the bandwidth below −10 dB is about 2.2 GHz. This phenomenon is due to the fact that dielectric loss and magnetic loss are improved at the same time as the increasing of concentration of FeCl_3_. However, as the concentration of FeCl_3_ reaches to 1.00 mol·L^−1^, the value of RL of the composite begins to decrease. This phenomenon is due to the aggregation of α-Fe and Fe_3_C particles, which leads to the reduction of dielectric loss and magnetic loss of the composite. The maximum RL frequency *f* can be expressed by the following equation [24]:

(3) $$f=c/4d\sqrt{\left|\mu_{r}\right|\left|\varepsilon_{r}\right|},$$

where *d* is the thickness. Therefore, the maximum RL frequency shifts to a lower frequency with an increasing thickness of the absorber, as shown in Figure 7.

In the future, the research of influence of the pore structure of biomass and carbonization temperature on the absorption properties of magnetic metal nanoparticles/woodceramic composite will be carried out.

### 3.5. Mechanical Properties

**Figure 8:**
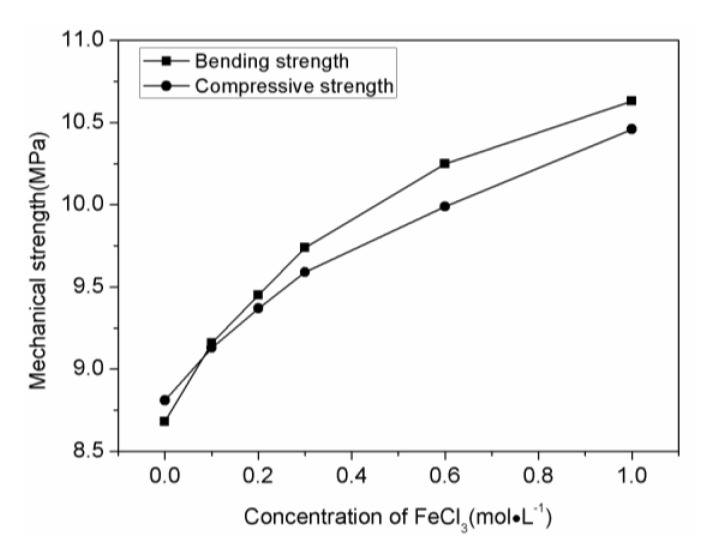
The mechanical strength of α-Fe/Fe_3_C/woodceramic nanocomposites as a function of concentration of FeCl_3_.

**Figure 9:**
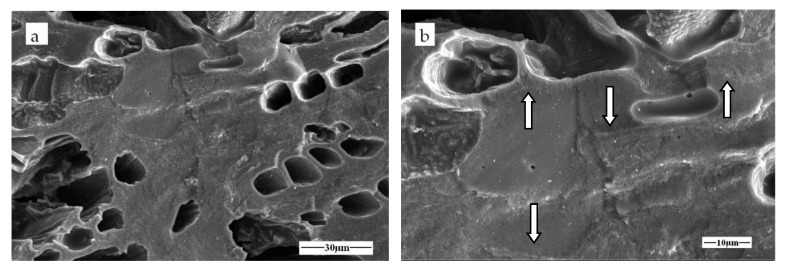
Morphology of the fracture surface of WCM-1.0 (**a**) Low-magnification SEM image; (**b**) High-magnification SEM image 3 mm.

Figure 8 shows the mechanical strength of α-Fe/Fe_3_C/woodceramic nanocomposites as a function of concentration of FeCl_3_. It can be seen that as the increasing of concentration of FeCl_3_, both the bending strength and compressive strength of α-Fe/Fe_3_C/woodceramic nanocomposites improve. The maximum value of bending strength of the composite reaches to 10.63 MPa when the concentration of FeCl_3_ is 1.00 mol·L^−1^, increasing by 2 MPa comparing to that of woodceramic, while the compressive strength of the composite reaches to 10.46 MPa, increasing by 1.70 MPa comparing to that of woodceramic. The enhancement of mechanical properties is due to the following two reasons. One is the improvement of extent of graphitization of the woodceramic, the other is the reinforcement effect of α-Fe and Fe_3_C nanoparticles on the woodceramic. According to the wall-bending model [25], the cracks are initiated at the wall consisting of wood layer and resin layer. It can be found from Figure 9a that the main wood structure has been remained in good shape in the composite. Figure 9b shows that glassy carbon has filled into some pores originated from the wood structure, the surface of which looks smooth. The wall as the arrows pointed looks rough, which indicates that α-Fe and Fe_3_C nanoparticles mainly disperse there. In the FeCl_3_ impregnation process, Ferric ions penetrate into the macro-pores and meso-pores of wood powder. α-Fe and Fe_3_C nanoparticles in-situ generate where is just the wall after sintering. These nanoparticles can hinder the initiation and propagation of the cracks, therefore the wall is strengthened.

## 4. Conclusions

A new α-Fe/Fe_3_C/woodceramic nanocomposite was fabricated. α-Fe and Fe_3_C nanoparticles are in-situ generated by carbothermal reduction reaction and combination reaction, respectively. These nanoparticles disperse in the matrix of woodceramic. The concentration of FeCl_3_ solution has great effect on the size of α-Fe and Fe_3_C particles. α-Fe nanoparticles have catalytic graphitization effect on woodceramic. α-Fe and Fe_3_C nanoparticles increase the dielectric loss and magnetic loss of woodceramic at the same time and consequently improve the absorption properties of woodceramic. WCM-0.6 shows the best absorption properties. The maximum value of RL at 3 mm reaches −25.60 dB at 10.16 GHz and the bandwidth below −10 dB is about 2.5 GHz. The mechanical properties of α-Fe/Fe_3_C/woodceramic nanocomposites are also improved comparing to that of woodceramic.
